# Integrating Multiscale Geospatial Environmental Data into Large Population Health Studies: Challenges and Opportunities

**DOI:** 10.3390/toxics10070403

**Published:** 2022-07-20

**Authors:** Yuxia Cui, Kristin M. Eccles, Richard K. Kwok, Bonnie R. Joubert, Kyle P. Messier, David M. Balshaw

**Affiliations:** 1 Division of Extramural Research and Training, National Institute of Environmental Health Sciences (NIEHS), National Institutes of Health (NIH), Durham, NC 27709, USA; yuxia.cui@nih.gov (Y.C.); bonnie.joubert@nih.gov (B.R.J.); 2 Division of the National Toxicology Program, National Institute of Environmental Health Sciences (NIEHS), National Institutes of Health (NIH), Durham, NC 27709, USA; kristin.eccles@nih.gov (K.M.E.); kyle.messier@nih.gov (K.P.M.); 3 Office of the Director, National Institute of Environmental Health Sciences (NIEHS), National Institutes of Health (NIH), Durham, NC 27709, USA; richard.kwok@nih.gov

**Keywords:** exposome, geospatial technologies, data integration, population health

## Abstract

Quantifying the exposome is key to understanding how the environment impacts human health and disease. However, accurately, and cost-effectively quantifying exposure in large population health studies remains a major challenge. Geospatial technologies offer one mechanism to integrate high-dimensional environmental data into epidemiology studies, but can present several challenges. In June 2021, the National Institute of Environmental Health Sciences (NIEHS) held a workshop bringing together experts in exposure science, geospatial technologies, data science and population health to address the need for integrating multiscale geospatial environmental data into large population health studies. The primary objectives of the workshop were to highlight recent applications of geospatial technologies to examine the relationships between environmental exposures and health outcomes; identify research gaps and discuss future directions for exposure modeling, data integration and data analysis strategies; and facilitate communications and collaborations across geospatial and population health experts. This commentary provides a high-level overview of the scientific topics covered by the workshop and themes that emerged as areas for future work, including reducing measurement errors and uncertainty in exposure estimates, and improving data accessibility, data interoperability, and computational approaches for more effective multiscale and multi-source data integration, along with potential solutions.

## 1. Introduction

The exposome, which is defined as the totality of an individual’s environmental exposure from conception onwards [1], has been increasingly adopted by the biomedical research community since Chris Wild’s initial commentary in 2005 [2,3]. Since that time, several large international research initiatives have been launched which have holistically collected and utilized genetic, environmental, lifestyle, and social and societal factors to better understand human health and disease [4,5,6,7]. In the United States, large and geographically distributed cohorts such as the *All of Us* Research Program [8], a diverse prospective cohort that will ultimately consist of one million participants across the U.S., and the Environmental Influences on Child Health Outcomes (ECHO) Program [9], which brings together separate cohorts to pool their data, provide unique opportunities to understand the health impacts of diverse environmental exposures. The ability to quantify an individual’s exposome and incorporate those measurements into the understanding of health and disease is key to precision health and personalized intervention and prevention. However, comprehensively assessing an individual’s exposome in large population studies remains a major challenge due to the broad range of environmental exposures and the variation through space and time.

The National Institute of Environmental Health Sciences (NIEHS) has been at the forefront of accelerating scientific and technological advancements to characterize the exposome. Focused efforts that address the exposome and personalized exposure assessments began even before Chris Wild’s initial 2005 commentary and continued with the establishment of the Exposure Biology Program within the Genes, Environment, and Health Initiative [10]. The launch of the Human Health Exposure Analysis Resource (HHEAR; previously, the Children’s Health Exposure Analysis Resource, or CHEAR) has provided centralized, scalable and harmonizable environmental exposure data by analyzing environmental chemicals and metabolites in biospecimens and environmental samples collected in population studies [11,12]. The exposome, however, encompasses not only exposures that can be measured in biological samples but also broad chemical and non-chemical factors that can be measured outside of the laboratory, such as air pollution, psychosocial stress, social determinants of health, and the built environment. Therefore, a comprehensive understanding of the exposome requires the integration of approaches and methodologies from a variety of fields, including analytical chemistry, biology, statistics, and geographic information systems (GIS). Recent advances in geospatial technologies and environmental sensing, such as remote sensing, GIS, global positioning system (GPS) technologies, and community and personal monitoring, provide important opportunities for the integration of location-based environmental measurements at much higher spatiotemporal resolution and precision than single technology alone can provide, and this can be leveraged to understand the impact that the environment has on disease etiology, prevention, and intervention [13,14,15].

To promote the application of geospatial technologies in population health studies and address current challenges, the NIEHS hosted a workshop titled “Integrating Multiscale Geospatial Environmental Data into Large Population Health Studies” in June 2021 [16]. The workshop brought together scientists from a wide range of disciplines, including exposure science, geospatial technologies, population science, genomics and genetics, and data science to discuss how to improve exposome characterization by leveraging multiscale geospatial environmental data (across time, space, and exposure types) in large-scale population studies. The workshop consisted of state-of-science presentations on geospatial technologies, exposure modeling, data science, and data integration, followed by panel discussions on challenges and research gaps. This commentary will provide a brief overview of the scientific discussions at the workshop and summarize potential future directions to advance the science.

## 2. Opportunities for Applying Geospatial Technologies to Advance Health Research

The workshop started with presentations centered on how geospatial technologies are used to characterize environmental exposures, including air and water contamination and social and neighborhood factors. Specifically, in regard to geospatial technologies to improve air pollution measurements, there have been various novel approaches and data sources to provide spatially and temporally resolved measurements that can be used to obtain exposure estimates. These approaches include satellite remote sensing, mobile monitoring, dense deployments of stationary low-cost sensors, and wearable technologies. Due to their complementary nature, when used in a combined fashion, these technologies provide a better understanding of temporal and spatial variation, thus reducing exposure measurement error and increasing the statistical power to detect relevant exposure–health associations. 

### 2.1. Satellite Remote Sensing 

Earth-observing satellites that generate raster-based remotely sensed data have become a powerful large-scale and low-cost tool for assessing population-level exposures to air pollutants (e.g., particulate matter (PM), ozone, NO_2_, and CH_2_O) and other environmental variables such as green space, walkability, light at night, harmful algal blooms, and noise. For decades, satellite products have been used in conjunction with ground-based monitoring, chemical transport models, and geostatistical methods to improve the spatial and temporal resolution and coverage of air pollution estimates, especially in regions where regulatory monitoring networks are sparse [17,18]. Exciting new National Aeronautics and Space Administration (NASA) missions, including Tropospheric Emissions: Monitoring of Pollution (TEMPO) and Multi-Angle Imager for Aerosols (MAIA), will continue to provide high-quality data on air pollutants [19,20]. These large-scale satellite-based methods (e.g., 250 m to 1 km resolution) are useful for population-level exposure estimates. Historically, these large-scale satellite-based datasets have been hard to use, and it is critical to make them more accessible and user-friendly to increase the utility to a wider audience. To address this challenge, resources such as the NASA Applied Remote Sensing Training Program (ARSET) are now available, which offers webinars and online courses with hands-on guided computer exercises on how to access and use NASA satellite datasets and analysis tools [21]. Applications of satellite air pollutant estimates were demonstrated using the NIEHS Sisters Study, where increased PM_2.5_ and NO_2_ exposure was associated with high blood pressure [22]. Outdoor light at night exposure, derived from satellite images, has been linked to increased breast cancer and thyroid cancer in the NIH AARP Diet and Health Study cohorts [23,24]. Increasing “greenness” was associated with a decrease in all-cause mortality in the Nurses’ Health Study [25].

### 2.2. Hyperlocal Mapping

Localized methods for quantifying exposure to air pollutants or neighborhood-level characteristics were also discussed, including mobile air monitoring in urban areas, dense deployment of low-cost stationary sensors at a neighborhood scale, and street view images for capturing multiple aspects of the neighborhood environment [26,27,28,29,30]. Technological advancement and cost efficiency in these methods have made it more feasible to generate a local exposure map with a much higher spatial and temporal resolution. There have also been interesting new opportunities to utilize citizen science to increase the number of localized monitors in a monitoring network or use crowdsourcing to expand data collection efforts. These localized monitoring data are often paired with other larger scale data, such as satellite images and advanced computational models, including machine learning, neural networks, and deep learning methods, to develop a more accurate and continuous map for a particular exposure [31,32]. Integrating the mobility and time-activity patterns captured by smart devices with satellite-derived data on the concentration of pollutants can better characterize individual microenvironments and obtain more accurate exposure concentrations, which may differ based on location (e.g., near a road vs. in a park) as well as activity (e.g., heavy breathing, such as during exercise, increases the volume of air inhaled). More precise estimates of exposure to pollutants can improve our understanding of their associations with other health measurements. This is a significant improvement in exposure assessment, compared to satellite data alone, which can only provide aggregate exposure estimates with lower spatiotemporal resolution. Localized exposure mapping can also be utilized for estimating chemical contaminants using vector-based GIS methods. Here, point measurements of contaminants from an environmental sample are geotagged with GPS coordinates and represent a discrete location in space and time. Examples include characterizing human exposure to various chemicals (e.g., arsenic, nitrates and PFAS) in public and private drinking water sources in the United States given the location of the well, a chemical analysis of the water sample, and information on well utilization [33,34]. Geolocated point estimates of chemical exposures can also be spatially linked to health outcome data. For example, this approach was used to identify high rates of bladder cancer among women who drank water with nitrates in the Women’s Health Study of Iowa [33]. However, an important aspect of accurately quantifying exposure–outcome relationships is to estimate the dose and duration of the exposure accurately, which can be challenging for longitudinal studies. Furthermore, understanding neighborhood-level behaviors and time–activity patterns through smart technologies, such as GPS-enabled smartphones or wearable activity trackers, may help inform more accurate personalized estimates of exposure overtime [35].

### 2.3. Personal Monitoring 

Personal environmental measurement captures exposure levels in the immediate proximity of a person and enables more accurate exposure estimation. Personal monitoring has become more accessible with recent advancements in wearable technologies. There is a wide array of wearable sensors available at relatively low cost that can measure various environmental factors including air pollution (e.g., PM, ozone, and toxic gases), UV, noise, temperature, physical activity, and physiological parameters (heart rate, blood pressure, ventilation, and body temperature) [36]. GPS data collected by wearable devices and smartphones provides another source of information on individual mobility patterns, which can be combined with large-scale exposure data (e.g., air pollution, green space) for more accurate exposure estimates at a personal level. Mobile phone applications (e.g., Ecological Momentary Assessment (EMA)) have been used in health studies to provide a contextual understanding of personal exposure. The Biomedical Real-Time Health Evaluation (BREATHE) informatics platform developed by the Los Angeles PRISMS Center is a great example of multi-sensor systems for characterizing how a person’s microenvironment drives adverse health effects [37]. There has also been an increasing adoption of wearable passive silicone samplers for capturing a wide range of volatile and semi-volatile chemicals in the personal environment, including polycyclic aromatic hydrocarbons (PAHs), pesticides, phthalates, and more [38].

## 3. Challenges, Research Gaps, and Research Advancements

Speakers at the workshop presented numerous new and emerging geospatial data sources and novel approaches for obtaining and applying location-based exposure measurements in health-related studies. Significant challenges and research gaps were discussed through presentations and panel discussions. Several crosscutting issues that need to be addressed emerged under two broad categories: (1) how to improve the accuracy of exposure estimates in geospatial analysis; and (2) how to enable data integration across multiple data modalities. 

### 3.1. Improving the Accuracy of Exposure Estimates by Reducing Measurement Errors and Controlling Uncertainty

Measurement errors and uncertainties can arise from multiple sources in exposure modeling such as exposure aggregation, missing covariates, and failure to account for time–activity patterns and other personal behaviors and characteristics. Several approaches were discussed to address the sources of measurement errors and to control uncertainty. 

#### 3.1.1. Model Validation against Independent Measurements

Spatial–temporal exposure modeling, which is the process of estimating an exposure concentration for an individual or aggregate group of individuals (i.e., census tract), is an important method for generating exposure estimates in locations and time periods where real exposure measurements are not available. For example, only a third of US counties have one or more EPA air monitors, leaving many small towns and rural areas with no air monitoring and no information on air quality due to the cost limitation [39]. Satellite-derived air quality data fill these important data gaps; exposures can be estimated using advanced modeling approaches using satellite aerosol optical depth (AOD) data, land use and meteorology data, and EPA ground monitoring networks. It is critical that the models used are validated against real measurements that are external to the model development, such as datasets from other sources, including crowd-sourced data and data collected through low-cost sensor monitoring networks. In areas where ground measurements are not available (e.g., in some developing countries), model validation becomes particularly challenging. This can be addressed by conducting validation studies by collecting real exposure measurements from a subset of larger studies to provide an alternative approach to addressing concerns over model validity, and subsequently help reduce measurement errors and improve data interpretation through model calibration against these measurements. This can be useful in population studies for many exposures that involve complex modeling, or when not all covariates can be easily incorporated into the model. 

#### 3.1.2. Incorporation of Mobility and Time–Activity Patterns

Measurement errors are a significant challenge in longitudinal exposure assessments. This is due not only to the difficulties of validating historical location-based estimates against available measurements, but also challenges in knowing individual mobility patterns within the timeframe, which can be decades, such as in cancer studies. Building complete residential histories is important, but not sufficient, as people spend many hours outside of their residence addresses at school and work. Mobile-based GPS data and agent-based modeling are promising approaches to address this data gap and provide better information on exposures over space and time which often can be misaligned. In large population studies, it is often not feasible to gather time–activity data on all participants. However, it may be possible to model more individualized exposures in a subset of study participants and use that information to build predictive algorithms for behavior and time–location patterns for the larger cohort, enabling the calibration of exposure estimates. Accounting for individual behavior and time–activity patterns and incorporating that information in exposure modeling is key to achieving more accurate and complete exposure estimates from the natural, social, and built environment. However, more research is needed in this area, and consideration must be given to protect privacy when individual-level time–location data are collected, shared, and used in exposure modeling so that stigma and discrimination can be prevented. 

#### 3.1.3. Data Gaps in Indoor Exposure

Most geospatial exposure models quantify outdoor chemical concentrations and exposure levels, but Americans, on average, spend approximately 90 percent of their time indoors [40]. The lack of data on indoor environments is a major limitation for geospatial exposure modeling. For example, in air pollution, multiple factors that impact indoor air quality need to be understood, including indoor sources that contribute to air pollutant concentrations, building characteristics that may impact penetration coefficients of outdoor pollutants, and individual behaviors. This gap can be addressed with data generated from personal sensors or home-based stationary sensors that provide real measurements of the pollutants [41]. For other non-airborne exposures, home environmental sampling, such as house dust, may help better elucidate indoor source and exposure level [42]. Overall, more research is needed to better characterize exposure to indoor pollutants and develop models that connect outdoor exposures to indoor exposures to create more complete exposure estimates. Recently, the National Academies of Sciences, Engineering, and Medicine released a report titled “Why Indoor Chemistry Matters” to call for further research in this area [43].

#### 3.1.4. Combining the Strengths of Diverse Geospatial Technologies

It is evident that no single data type or technology can provide both the comprehensive coverage and the level of spatial and temporal resolution that are desired for human health research. One of the ways forward is to combine different geospatial technologies that provide information at different spatial and temporal scales. There is tradeoff for using each data type individually, while integrating methods that have different spatial, and temporal resolution can help to develop more accurate and cost-effective exposure models. We exemplify this in Table 1 using air pollution assessments; a wide array of geospatial exposure assessment technologies and approaches have been developed in recent years which provide dense and spatially resolved exposure data. These include wearable sensors, community low-cost sensor networks, and mobile monitoring. Exposure modeling can leverage these different data streams and combine them with satellite remote sensing to develop better predictive models for more accurate exposure assessments at an individual level. Exposure data enabled by diverse technologies also provide opportunities for model validation against independent measurements. 

### 3.2. Enabling Multiscale Data Integration by Improving Data Access and Computational Methods and Models

There has been a dramatic increase in the amount of publicly available geospatial datasets in the last two decades, attributed to advances in ubiquitous environmental sensing, GIS technologies, and crowdsourcing. Yet, the utility of these datasets has not been fully utilized for health research. This is partly because many geospatial datasets are not easily findable, accessible, interoperable, or reusable by general health researchers (the FAIR principles) [44]. Additionally, integrating multiscale and diverse geospatial environmental data with complex personal health outcome data requires advanced computational methods, models, and ethical considerations to protect participant privacy, as well as interdisciplinary collaborations. The section below will discuss challenges in data access, computational methods and models, and potential solutions. 

#### 3.2.1. Data Access and Data Interoperability

There are numerous publicly available geospatial datasets, but many of them are not easily accessible or readily usable by health researchers. In many cases, data science expertise is required to obtain and utilize these data. For example, data transformation and exposure modeling may be needed to convert satellite imagery data to air quality estimates before it can be applied in health research, which requires not only proficiency in computer programming languages but expertise in atmospheric science. There are also multiple datasets on the same pollutants generated using different exposure modeling approaches and with different spatiotemporal coverage, which creates further confusion for non-expert users. Through partnering with epidemiologists and health organizations, the new NASA MAIA mission, which will be launched in the near future, will produce air quality data that can be used directly by the health research community [45]. These include total PM_10_ and total PM_2.5_, as well as PM_2.5_ speciation. This will greatly improve the accessibility of the new data by the health research community. For existing diverse geospatial datasets (such as historical air monitoring, water contamination, pesticide usage, and administrative data), proper documentation on how the data were generated, including the advantages and limitations of each exposure modeling approach, will help guide the selection of the right datasets for the research question and improve data interoperability and integration. This would require collaborative efforts across the global science community to develop and promote common data standards and metadata standards.

#### 3.2.2. Data Infrastructure and Data Platforms

Data infrastructure and data platforms are critical for promoting data sharing and data integration. Establishing and maintaining such infrastructure needs substantial involvement from the scientific community. The Canadian Urban Environmental Health Research Consortium (CANUE) provides an example of how a centralized data platform can work. The CANUE DATA PORTAL not only provides researchers access to large-scale, historical geospatial datasets, but a set of statistical and data science tools to facilitate data analysis and integration [46]. Another example of a centralized platform is the geospatial resource established by the NIH Environmental Influences on Child Health Outcomes (ECHO) Program. This brings together a diverse set of geospatial data, methods, and modeling approaches to allow consortium researchers to look at the effects of environmental and social risk factors in a nationwide, geographically diverse cohort [9]. Currently, building geospatial data infrastructures and data platforms around large consortia seems to be an efficient way to support geospatial data sharing and integration. It was also recognized by the workshop participants, however, that the community should encourage broader sharing and the utilization of datasets and data science tools to prevent duplicative efforts. 

#### 3.2.3. Data Analysis across Multiple Modalities

Accurate and efficient data integration of multiscale and diverse data across environment, genetics and health outcomes is essential for maximizing the utility of geospatial datasets for research. However, it remains a significant challenge in environmental health studies. A common challenge is how to disentangle the highly correlated exposure measures and confounding variables in exposure–health association analysis. A number of new statistical methods developed by the NIEHS Powering Research Through Innovative Methods for Mixtures in Epidemiology (PRIME) Program have been published recently, including several new methods addressing mixtures of exposures that vary over space and time [47]. In addition to statistical strategies, data science methods such as machine learning and artificial intelligence (AI) have been increasingly applied in the analyses of complex environmental health data for exposure prediction, disease prediction, and causal inference [48]. Another significant challenge is the integration of multi-dimensional and time-varying geospatial environmental data with high dimensional omics data, such as metabolomics, genomics, and epigenomics data. It is becoming increasingly apparent that health outcomes are the result of complex interactions between genetic variations and complex environmental exposures that impact common biological pathways implicated in many diseases. While comprehensive exposure measurements and high-dimensional omics data together offer the opportunity to study both mediation and more complex gene environment interactions, the true understanding of complex biological systems requires not only advanced computational approaches, but also the incorporation of biological knowledge into the data analysis. Last but not least, a common barrier in data analysis is how to scale up the linkage of spatial data to personal health information while protecting participant privacy. The confidentiality of patients and research subjects must be safeguarded. DeGAUSS (Decentralized Geomarker Assessment for Multi-Site Studies), a software application developed for multi-site studies, provides a method for decentralized geocoding to avoid the translocation of sensitive participants’ residential data from one site to another [49]. 

## 4. Conclusions

The complex nature of the exposome requires an interdisciplinary approach to implement in health studies. Population-based cohort studies offer several strengths including the measurement of exposure prior to disease onset, stored biospecimens, robust covariate information, and the opportunity for follow-up and repeated sampling. Additionally, the geographic and genomic variation that cohort studies can provide make it a particularly attractive resource for researchers. Therefore, existing cohorts that have rich longitudinal data such as physical measurements, biologic information, questionnaire data, and up-to-date information from participants’ electronic health records (EHR) should be leveraged. Opportunities need to be created for geospatial experts to collaborate with cohorts to identify important scientific questions, bring expertise together, and develop use cases where new research questions can be addressed by incorporating geospatial datasets. The diverse demographics and health outcomes of large national and international cohorts may offer more power to discover geographically linked exposures impacting health, especially for rare exposures and diseases. For example, the *All of Us* Research Program is a large nation-wide longitudinal cohort that aims to collect electronic health records, self-reported survey data including geographic location, physical measurements, bio-samples, genetic and digital health data on participants ages 18 and above. *All of Us* has been designed as a platform program which is disease agnostic and will allow researchers to utilize data to answer their own questions without worrying about recruitment issues. Integrating multiscale geospatial environmental data into large population health studies such as *All of Us* presents a unique opportunity to better understand how environmental exposures can impact health on a local scale. Moving forward, communication is the first step to bridge the disconnection between research communities (exposure science, geospatial technologies, population science, genomics and genetics, and data science). International coordination on data and metadata standards and exposure modeling efforts is key to promoting broader sharing and the better utility of geospatial data and resources. The NIEHS Environmental Health Language Collaborative is an initiative to advance community development and the application of a harmonized language for environmental health [50]. This is an ongoing effort to address challenges in data harmonization and interoperability, including placed-based measurements. Furthermore, federated data platforms can provide easy access to implementable datasets and interoperability across studies. It is also important to build diversity into the organizing structure of large initiatives so that appropriate expertise in environmental health science will be included and environmental factors will be considered at the planning stage of large longitudinal cohorts. 

## Figures and Tables

**Table 1:** Comparison of diverse geospatial technologies and data types discussed at the workshop (using air pollution (PM_2.5_, NO_2_, O_3_, etc.) as an example).

	Satellite Remote Sensing	Hyperlocal Mapping	Personal Monitoring
**Spatial and Temporal Coverage**	Global or large geographical area; years to decades of data	Neighborhood or community; months to years of data	Individual; usually days to weeks of data
**Spatial and Temporal Resolution**	Varies across measurements, and usually low (250 m–1 km or lower); annual or daily average	Street level (10–30 m); multiple time points per day or real time	Immediate proximity of the person; real time (minutes or seconds)
**Ambient or Indoor**	Ambient measurements only	Ambient measurements only	Both indoor and outdoor measurements
**Cost**	Publicly available data, no cost to the users	May require new data collection, cost to the user is medium	Likely requires extensive efforts for data collection, cost to the user is high
**Disadvantages**	Lower resolution of data may not be sufficient, and pollutants are limited	Require modeling techniques and validation to make the point estimates into useable continuous surfaces	Cost to collect, store, and analyze the highly dimensional dataset is high
